# From arrest to circuit: Mapping twenty years of progress and pitfalls in ECPR implementation, patient selection, and initiation timing☆

**DOI:** 10.1051/ject/2026019

**Published:** 2026-09-15

**Authors:** Nousjka P.A. Vranken, Patrick W. Weerwind, Raymond K. Wong

**Affiliations:** 1 Department of Cardiology, Zuyderland Medical Center Heerlen the Netherlands; 2 CTRC Vascular & Medical Devices Wijchen the Netherlands; 3 Perfusion Sciences, Department of Pharmacology, College of Medicine–Tucson, The University of Arizona Tucson AZ United States

**Keywords:** Extracorporeal cardiopulmonary resuscitation, Outcomes, Extracorporeal life support

## Abstract

*Background*: Although the literature on extracorporeal cardiopulmonary resuscitation (ECPR) has grown substantially, coherent integrative paradigms remain limited. This study synthesizes two decades of research to elucidate knowledge gaps and orient future inquiry. *Methods*: A mapping review of studies from 2004 to 2024 was performed via PubMed and EMBASE. Only studies comparing ECPR to conventional cardiopulmonary resuscitation were included. Data were extracted and synthesized thematically. *Results*: A total of twenty studies were included, the majority being retrospective and observational design, including three interventional studies. Mortality rates following ECPR remain high and more or less constant, up to 86%. To improve chances of survival, several factors need to be considered. First is patient selection: age limit, initial cardiac rhythm, bystander resuscitation with sufficient efficacy, and maximum low-flow time are common criteria, although inconsistently applied. Some studies suggest higher ECPR survival rates in specific clinical scenarios, but none of the previous studies have yet succeeded in formulating an algorithm that facilitates the identification of the most suitable candidates. The second factor affecting outcomes is the pathophysiologic processes during ECPR. This is largely unexplored territory, with current knowledge limited to observational studies of low-flow injury. Most studies do not report on factors including temperature management during support, pump flow, target mean arterial pressures, and oxygenation strategies. Lastly, and potentially most significantly, is the timing of ECPR initiation. Timely restoration and maintenance of circulation and oxygenation halt the accumulation of oxygen debt and commence oxygen debt repayment. To date, data on optimal initiation timing thresholds remains undetermined. *Conclusion*: Current ECPR literature lacks knowledge across three interdependent domains that require an integrated approach: validated selection criteria, a mechanistic understanding of ischemia-reperfusion injury, and precise initiation-timing strategies. A multidisciplinary research agenda is needed to unlock the true value of ECPR in cardiac arrest care.

## Introduction

Cardiovascular disease remains the leading cause of mortality worldwide. Survival following cardiac arrest – regardless of underlying etiology – remains low, with rates of approximately 25% for in-hospital cardiac arrest (IHCA) and 10% for out-of-hospital cardiac arrest (OHCA), with even lower rates of neurologically intact recovery [1, 2].

Extracorporeal cardiopulmonary resuscitation (ECPR) is a complex, resource-intensive intervention that restores and maintains circulation in the setting of cardiac arrest. It is considered in cases of refractory cardiac arrest, commonly defined as persistent arrest after 15 min of conventional cardiopulmonary resuscitation (CCPR) [3]. By sustaining systemic oxygenation and circulation, ECPR provides a critical window during which clinicians can identify and treat the underlying cause of arrest. Following its first successful clinical use in the 1960s, ECPR regained global attention during the H1N1 influenza pandemic in 2009 [4].

Despite growing interest, with the number of ECPR-related publications tripling over the past decade, robust evidence of a meaningful impact on neurologically intact survival remains limited. Registry data indicate survival rates following ECPR as low as 30%, with fewer than 20% achieving favorable neurological outcomes [5, 6]. Multiple observational studies – and only a limited number of prospective controlled trials – have evaluated ECPR, predominantly in small OHCA cohorts with presumed acute coronary syndrome. In a considerable proportion of cases, the etiology of cardiac arrest is unknown at the time of intervention.

Two randomized controlled trials have provided important insights into the potential benefits of ECPR. The ARREST trial compared ECPR to CCPR in refractory OHCA due to ventricular fibrillation/tachycardia, reporting a substantial survival-to-discharge benefit: 43% in the ECPR arm versus 7% in CCPR, even leading to early trial termination [7]. The Prague OHCA study, including non-shockable rhythms, randomized 256 patients to a hyperinvasive strategy (transport with mechanical CPR, catheterization, and ECPR) versus CCPR. Neurologically intact survival at 30 days was significantly higher in ECPR, 30.6% versus 18.2% (*p* = 0.02) [8]. Importantly, extended resuscitation beyond 30 min showed a clear benefit only in the invasive arm; 28% favorable outcome compared to 7.6% in CCPR [9]. Subgroup analyses highlighted that initial shockable rhythms, shorter low-flow times, and younger age predict better outcomes [10]. Conversely, pulmonary embolism-related OHCA showed poor neurological outcomes compared to other etiologies despite ECPR (8.3 vs 28.4%, respectively), underscoring the importance of etiology-specific patient selection and management [11].

Recent reviews have questioned the overall benefit of ECPR, proposing it to be reserved as a last-resort treatment modality in carefully selected cases [12, 13]. Another meta-analysis offers a more optimistic perspective, indicating a doubling in favorable neurological outcomes with ECPR compared to CCPR (14% vs 7%, respectively) and improved overall survival [14, 15]. Due to the lack of studies focusing on specific patient subsets defined by arrest etiology, as well as the absence of tailored ECPR management strategies, we hypothesize that the full potential of ECPR, as of now, remains unrealized. Since bridging knowledge gaps is essential for optimizing outcomes and resource utilization, we aim, through this mapping review, to reveal modifiable factors that could be deployed as leverage points in future research.

## Methods

### Literature search strategy

A comprehensive search of the PubMed and EMBASE databases was conducted to identify studies published between March 2004 and March 2024 comparing outcomes between CCPR and ECPR. The primary outcomes of interest were overall survival and neurologically intact survival. The search strategy combined medical subject headings (MeSH) and free-text terms related to “ECPR”, “extracorporeal resuscitation”, “ECMO” – extracorporeal membrane oxygenation, “cardiac arrest”, “conventional CPR”, and “neurological outcome”. Only studies in English and involving adult populations (≥18 years) were included. Articles involving pediatric populations or published in languages other than English were excluded.

### Study selection

**Figure 1:**
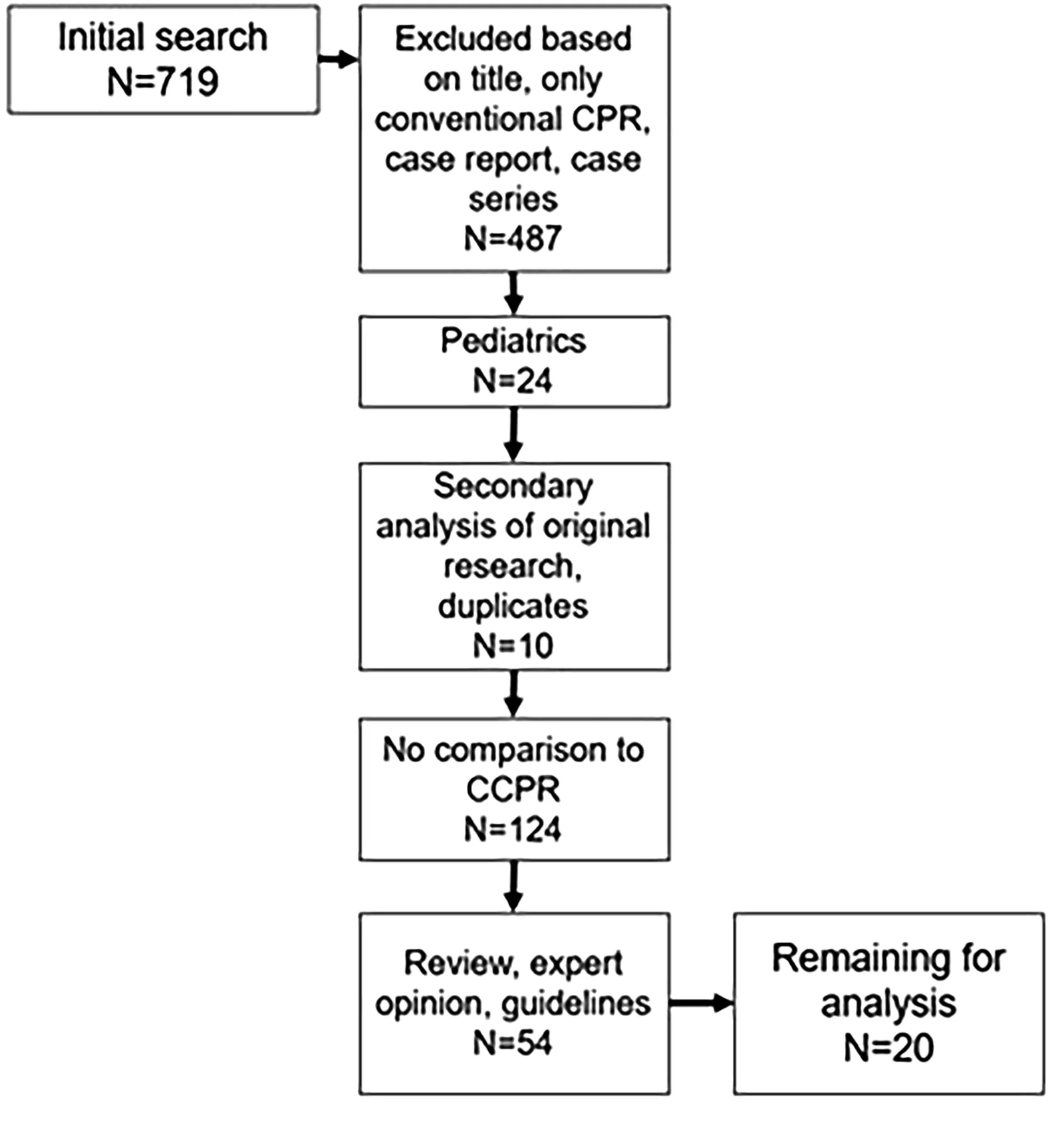
Data collection flow diagram.

All retrieved records were screened by title and abstract by two independent researchers (NV and PW). Full-text articles were reviewed for eligibility based on predefined inclusion criteria. Discrepancies were resolved through discussion or, when necessary, by consulting a third reviewer. A Preferred Reporting Items for Systematic Reviews and Meta-Analyses (PRISMA) flow diagram (Figure 1) summarizes the study selection process, including reasons for exclusion at each stage.

### Data extraction

From each included study, the following variables were extracted:

- Study characteristics: date of data collection and number of participating centers.
- Population characteristics: sample size of ECPR and CCPR groups, mean or median age, proportion of female patients, OHCA cases, proportion of unwitnessed arrests, and proportion of shockable initial rhythms.
- ECPR-specific parameters: time from arrest to ECPR initiation, ECPR flow rate, mean arterial pressure (MAP), arterial partial pressure of oxygen (PaO_2_), and use of targeted temperature management during ECPR. Targeted temperature applied as part of general post-resuscitation care (not specific to ECPR) was not included in the current analysis, as this is routinely applied across both ECPR and CCPR cohorts [16].

## Results

A total of 20 studies were included in this review. The majority employed retrospective (*n* = 13) and observational (*n* = 17) study designs. Only seven studies were prospective, and three were interventional in nature [7, 8, 17].

Regarding patient selection, common exclusion criteria included: advanced age (typically >75 years), trauma mechanism responsible, either IHCA or OHCA, known pre-existing terminal illness, and significant neurological impairment. In addition, several studies excluded patients with an obvious non-cardiac cause of the arrest, unwitnessed arrest, or active, uncontrollable bleeding (Tables 1 and 2). Significant variability in inclusion and exclusion criteria exists across studies, notably in upper age limit (ranging from 50 to 80 years), bystander CPR status, initial cardiac rhythm, and (maximum) estimated low-flow times.

**Table 1:** Study characteristics of included retrospective studies.

Author	Maekawa [18]	Lin [19]	Shin^2013^ [20]	Kim [21]	Siao [22]	Patel [23]	Cesana [24]	Han [25]	Mandigers [26]	Patricio [27]	Shin^2020^ [28]	Bartos [29]	Choi [30]
Data collection year	2000–2004	2004–2006	2003–2009	2006–2013	2011–2013	2006–2014	2011–2015	2006–2017	2012–2017	2012–2017	2011–2019	2012–2019	2013–2020
Single/multicenter (N)	single	single	single	Single	single	multi (363)	Single	single	multi (2)	single	single	single	multi (700)
N ECPR	53	55	85	55	20	32499	63	25	19	112 (80 matched patients)	30	160	484
N CCPR	109	63	321	444	40	775	85	178	20	523 (80 matched controls)	40	654	117907
Age ECPR	54 (47–60)	59.0 (11.7%)	59.9 ± 15.3	53 (41–68)	54.55 (11.94)	majority (44.4%) is 45–64	59 ± 10		40 (30–60)	54 ± 16	60 (55–67)	57 ± 1.0	55.2 (14.2)
Age CCPR	71 (59–80)	60.6 (12.7%)	61.6 ± 14.2	69 (56–77)	60.28 (11.23)	majority (34.2%) is 45–64	63 ± 9	70 (61–79)(overall group)	56 (46–64)	65 ± 16	61 (51–70)	59 ± 0.4	70.1 (15.0)
Female (%) ECPR	9 (17%)	14.50%	32 (37.6%)	14 (25.4%)	2 (10%)	63.50%	8 (13%)		11 (58%)	29 (25.9%)	5 (16.7%)	34 (21%)	88 (18.2%)
Female (%) CCPR	30 (27.5%)	34.90%	120 (37.4%)	159 (35.8%)	12 (30%)	57.40%	21 (25%)	38.9%(overall group)	12 (60%	187 (35.8%)	10 (25%)	126 (19%)	43,296 (36.7%)
OHCA ECPR	53 (100%)	0	0	55 (100%)	11 (55%)	32499 (100%)	46 (73%)	–	13 (68)	70 (63%)	30 (100%)	100%	193 (39.9%)
OHCA CCPR	109 (100%)	0	0	444 (100%)	30 (75%)	775 (100%)	64 (75%)	–	8 (42%)	281 (54%)	40 (100%)	100%	20,118 (17.1%)
Unwitnessed ECPR	0	0	0	12 (21.8%)	–	–	0	–	–	19 (17%)	0	39% (24%)	128 (26.4%)
Unwitnessed CCPR	0	0	0	116 (26.1%)	–	–	0	33.5%(overall group)	–	136 (26%)	0	200 (30.6%)	58,537 (49.6%)
Shockable rhythm ECPR	32 (60.4%)	28 (50.9%)	25 (29.4%)	31 (56.4%)	20 (100%)	32.2%	40 (65%)		–	35 (32%)	18 (60.0%)	100%	292 (60.3%)
Shockable rhythm CCPR	24 (22.0%)	26 (41.3%)	73 (22.7%)	85 (19.1%)	40 (100%)	51.6%	61 (72%)	35 (17.2%)	–	141 (27%)	19 (47.5%)	100%	16,057 (13.6%)
Time arrest to ECPR (min)	49(41–59)(duration CPR) + 2 (0–8)(arrest to BLS)	–	CPR duration 42.1 ± 25.7	69 (median)	survivors: 56.5 (62.0) + 1–4 min before CPR / non–survivors: 41.5 (25.7) + 1–5 min before CPR	–	58 ± 25 (cardiac arrest time)	30 (21–44)(ACLS duration overall group)	77 (39–98) (CPR time) + 0 (0–2.25) (CPR delay time)	54 ± 22 (arrest to termination of CPR)	60.2 (18.9)	60 ± 1 duration of professional CPR	74.0 [61.0, 92.0]*
Targeted temp management ECPR	26(49.1%)	–	–	17 (30.9%)	9 (45%)	3.6%	57 (91%)	26 (12.8%)(overall group)	–	100%	22 (73.3%)	100%	83 (17.1%)
PO_2_ in ECPR (kPa)	–	–	–	–	–	–	–	–	–	80 – 150 mmHg	–	–	–
PCO_2_ in ECPR (kPa)	–	–		–	–	–	40–45 mm Hg	–	–	–	–	–	–
MAP during ECPR (mmHg)	sys 90	–	>65	MAP ≥60 within 2 h after ECPR *n* = 33 (75%)	–	–	–	⩾65	–	>65–70	–	–	–
Initial ECPR max flow (L/min)	50–60 mL/min/kg		2.2 L/min/m^2^	2.5–3.0 L (protocol)	–	–	2.5 l/min/m^2^	–		3–4	–	–	–
Details on system	available	available	partly available	available	available	–	partly available	–	not available	available	not available	available	–
IABP during ECPR	27 (50.9%)	–	–	–	10 (50%)	–	23 (38%)	–	–	–	–	–	–
Number of vasopressors/inotropes	1–2	–	–	2 (1–3)	–	–	–	–	–	–	–	–	–

CCPR: conventional cardiopulmonary resuscitation, ECPR: extracorporeal cardiopulmonary resuscitation, IABP: intra-aortic balloon pump, MAP: mean arterial pressure, OHCA: out-of-hospital cardiac arrest, PCO_2_: partial pressure of carbon dioxide, PO_2_: partial pressure of oxygen.

**Table 2:** Study characteristics of included prospective studies.

Author	Chen [31]	Chou [32]	Sakamoto [33]	Nakashima [34]	Belohlavek [8]	Yannopoulos [7]	Suverein [17]
Data collection year	2004–2006	2006–2010	2008–2011	2008–2011	2013–2020	2019–2020	2017–2021
singe/multicenter (N)	single	Single	multi (26 + 20)	multi (46)	single	single	multi (10)
Observational/Interventional	observational	Observational	observational	Observational	interventional	interventional	interventional
N ECPR	59	43	250	206	124	14	70
N CCPR	113	23	157	138	132	15	64
Age ECPR	57.4 (12.5)	60.5 ± 11.6	56.3	59 (48–64)	59 (48–66)	59 (10)	–
Age CCPR	60.3 (13.3)	69.6 ± 13.3	58.1	60 (51–68)	57 (47–65)	58 (11)	–
Female (%) ECPR	9 (15.3%)	3 (7%)	25 (9.6%)	23 (9%)	22 (18%)	1 (7%)	–
Female (%) CCPR	40 (35.4%)	6 (26%)	22 (11.3%)	18 (11%)	22 (17%)	4 (27%)	–
OHCA ECPR	0	0	260 (100%)	206 (100%)	124 (100%)	14 (100%)	–
OHCA CCPR	0	0	193 (100%)	138 (100%)	132 (100%)	15 (100%)	–
Unwitnessed ECPR	0	0	73 (28.5%)	68 (27%)	0	3 (21.4%)	2 (3%)
Unwitnessed CCPR	0	0	42 (22.2%)	36 (22%)	0	2 (13.3%)	1 (2%)
Shockable rhythm ECPR	29 (49.2)	26 (60.5%)	260 (100%)	127 (61.7%)	72 (58%)	14 (100%)	69 (99%)
Shockable rhythm CCPR	36 (31.9)	9 (39.1%)	193 (100%)	56 (40.6%)	84 (64%)	15 (100%)	63 (98%)
Time arrest to ECPR (min)	52·8 (37·2)	resuscitation time (36.6 ± 16.5) (survivors) 72.1 ± 37.8 min (nonsurvivors)	60**	54 (46–65)	61 (55–70)	59 (28)	–
Left ventricle decompression	3 (5.1)	–	–	–	–	–	–
Targeted temp management/hypoth ECPR	83 (17.1%)	–	162 (91.5%)	168 (89%)	117 (95%)	–	–
PO2 during ECPR (kPa)	–	–	–	–	–	–	8 (3–18)
MAP during ECPR	60 mmHg	–	65 mmHg or above, target diuresis 0.5 mL/h or more	–	–	–	–
PCO2		–	–	–	–	–	–
Initial ECPR max flow (L/min)	50–100 ml/kg/min, with adjustment of vasopressors	60 mL/kg/min	maximal flow rate (target: 4 L/min or above	2.0 (2.5–3.5)	–	–	–
Details on system	availlable	Available	–	–	not available	not available	–
IABP during ECPR	–	–	164 (92.7%)	173 (94%)	–	6 (40%)	20 (29%) (or impella)
Number of vasopressors/inotropes	3	–	–	–	–	–	–

CCPR: conventional cardiopulmonary resuscitation, ECPR: extracorporeal cardiopulmonary resuscitation, IABP: intra-aortic balloon pump, MAP: mean arterial pressure, OHCA: out-of-hospital cardiac arrest, PCO_2_: partial pressure of carbon dioxide, PO_2_: partial pressure of oxygen.

Information on the etiology of cardiac arrest was reported in some studies. While few studies provided diagnostic breakdowns, others presumed a cardiac origin without definitive confirmation (Tables 3 and 4). Elaboration on patient management strategies, i.e., target values for hemodynamic and blood gas parameters during ECPR, was lacking in most studies. The management strategy was described and not studied in or across subgroups. Eight out of twenty studies provided technical details regarding the extracorporeal membrane oxygenation (ECMO) circuits. Out of twenty studies, seven reported MAP during ECPR, and eight reported pump flow rates. The additional use of an intra-aortic balloon pump, as well as the doses (or equivalent dosing) of administered inotropes and vasopressors, was noted in seven studies. Only one study reported the use of left ventricular decompression, observed in 5.1% of ECPR patients [31]. Another study specified a targeted PaCO_2_ of 40–45 mmHg during support [24]. Targeted temperature management was applied in most studies, with the exception of two retrospective and three prospective studies providing no information on temperature management (Tables 1 and 2).

**Table 3:** Etiology of cardiac arrest in included retrospective studies.

Author	Maekawa [18]	Lin [19]	Shin^2013^ [20]	Kim [21]	Siao [22]	Patel [23]	Cesana [24]	Han [25]	Mandigers [26]	Patricio [27]	Shin^2020^ [28]	Bartos [29]	Choi [30]
ECPR: cardiac origin	100%	–	79 (92.9%)	49 (89.1%)	–	–	–	–	–	–	29 (96.7%)	100%	–
ECPR: non-cardiac origin	–	–	–	6 (10.9%)	–	–	–	–	–	42 (38%)	–	–	–
ECPR: ACS	–	36 (65.5%)	38 (44.7%	36 (69.2% of matched group *n* = 52)	12 (60%)	–	100%	–	–	–	–	–	–
ECPR: heart failure	–	4 (7.3%)	11 (12.9%)	3 (5.8%)	–	–	–	–	–	–	–	–	–
ECPR: pulmonary embolism	–	1 (1.8%)	4 (4.7%)	2 (3.8)	2 (10%)	–	–	–	100%	–	–	–	–
ECPR: arrhythmia	–	–	1 (0.2%)	3 (5.8%)	–	–	–	–	–	–	–	–	–
ECPR: valve disease	–	–	–	–	0	–	–	–	–	–	–	–	–
ECPR: myocarditis	–	3 (5.5%)	0	–	2 (10%)	–	–	–	–	–	–	–	–
ECPR: cardiomyopathy	–	–	–	–	2 (10%)	–	–	–	–	–	–	–	–
ECPR: respiratory arrest	–	–	2 (2.4%)	1 (1.9%)	–	–	–	–	–	–	–	–	–
ECPR: hypothermia	–	–	–	1 (1.9%)	–	–	–	–	–	–	–	–	–
ECPR: hypovolemia	–	–	4 (4.7%)	–	–	–	–	–	–	–	–	–	–
ECPR: AKI	–	–	–	1 (1.9%)	–	–	–	–	–	–	–	–	–
ECPR: brain hemorrhage	–	–	–	1 (1.9%)	–	–	–	–	–	–	–	–	–
ECPR: electrolyte/toxin	–	–	–	–	1 (5%)	–	–	–	–	–	–	–	–
ECPR: post–cardiotomy	–	7 (12.7%)	–	–	–	–	–	–	–	–	–	–	–
ECPR: unknown	–	3 (5.5%)	25 (29.4%)	4 (7.7%)	1 (5%)	32499 (100%)	–	–	–	–	100%	–	458 (100%)

CCPR: cardiac origin	100%	–	261 (81.3%)	277 (62.4%)	–	–	–	110 (54.2%)(overall goup)	–	–	–	–	–
CCPR: non–cardiac origin	–	–	–	167 (37.6%)	–	–	–	–	–	283 (54%)	–	–	–
CCPR: ACS	–	46 (73.0%)	82 (25.5%)	9 (17.3% of matched group *n* = 52)	16 (40%)	–	100%	–	–	–	52 (81%)	–	–
CCPR: heart failure	–	9 (14.3%)	48 (14.5%)	2 (3.8%)	–	–	–	–	–	–	–	–	–
CCPR: pulmonary embolism	–	0	5 (1.6%)	1 (1.9%)	6 (15%)	–	–	–	100%	–	–	–	–
CCPR: arrhythmia	–	–	0	5 (9.6%)		–	–	–	–	–	–	–	–
CCPR: valve disease	–	–	–	–	1 (2.5%)	–	–	–	–	–	–	–	–
CCPR: myocarditis	–	1 (1.6%)	1 (0.3%)	–	0	–	–	–	–	–	–	–	–
CCPR: respiratory arrest	–	–	28 (8.7%)	0	–	–	–	–	–	–	–	–	–
CCPR: hypothermia	–	–	–	0	–	–	–	–	–	–	–	–	–
CCPR: hypovolemia	–	–	32 (10.0%)	–	–	–	–	–	–	–	–	–	–
CCPR: AKI	–	–	–	3 (5.8%)	–	–	–	–	–	–	–	–	–
CCPR: brain hemorrhage	–	–	–	1 (1.9%)	–	–	–	–	–	–	–	–	–
CCPR: electrolyte/toxin	–	–	–	–	6 (15%)	–	–	–	–	–	–	–	–
CCPR: cardiomyopathy	–	–	–	–	4 (10%)	–	–	–	–	–	–	–	–
CCPR: post–cardiotomy	–	0	–	–	–	–	–	–	–	–	–	–	–
CCPR: unknown	–	7 (11.1%)	125 (38.9%)	31 (59.6%)	7 (17.5%)	775 (100%)	–	–	–	–	100%	100%	1832 (100%)

ACS: acute coronary syndrome, AKI: acute kidney injury, CCPR: conventional cardiopulmonary resuscitation, ECPR: extracorporeal cardiopulmonary resuscitation.

**Table 4:** Etiology of cardiac arrest in included prospective studies.

Author	Chen [31]	Chou [32]	Sakamoto [33]	Nakashima [34]	Belohlavek [8]	Yannopoulos [7]	Suverein [17]
ECPR: cardiac origin	55 (93.2%)	–	100%	183 (88.8%)	–	–	–
ECPR: ACS	37 (62.7%)	presumable 100%	165 (63.5%)	163 (65%)	64 (52%)	–	51 (73%)
ECPR: chronic CAD	–	–	–	–	14 (11%)	–	–
ECPR: heart failure	6 (10.2%)	–	–	–	8 (7%)	–	–
ECPR: pulmonary embolism	1 (1.7%)	–	–	–	12 (10%)	–	1 (1%)
ECPR: pulmonary hypertension	–	–	–	–	2 (2%)	–	–
ECPR: arrhythmia	–	–	42 (16.2%)	–	–	–	11 (16%)
ECPR: valve disease	–	–	–	2(1%)	–	–	–
ECPR: myocarditis	5 (8.5%)	–	2 (0.8%)	2(1%)	6 (5%)	–	–
ECPR: cardiomyopathy	–	–	17 (6.5%)	16 (7%)	3 (2%)	–	–
ECPR: hypothermia	–	–	–	–	3 (2%)	–	–
ECPR: sepsis	–	–	–	–	0	–	–
ECPR: brain hemorrhage	–	–	–	–	1 (1%)	–	–
ECPR: neurologic	–	–	–	–	–	–	0
ECPR: electrolyte/toxin	–	–	–	–	–	–	1 (1%)
ECPR: intoxication	–	–	–	–	–	–	1 (1%)
ECPR: post–cardiotomy	7 (11.9%)	–	–	–	–	–	–
ECPR: bleeding	–	–	–	–	3 (2%)	–	–
ECPR: type A aortic dissection	–	–	–	–	2 (2%)	–	–
ECPR: sarcoidosis/genetic mutation	–	–	–	–	–	–	1 (1%)
ECPR: unknown	3 (5.1%)	–	27 (10.4%)	67 (26%)	3 (2%)	100%	3 (4.3%)

CCPR: cardiac origin	–	–	100%	–	–	–	–
CCPR: ACS	80 (70.8%)	presumably 100%	115 (59.3%)	82 (52%)	63 (48%)	–	–
CCPR: chronic CAD	–	–	–	–	18 (14%)	–	–
CCPR: heart failure	18 (15.9%)	–	–	–	6 (5%)	–	–
CCPR: pulmonary embolism	0	–	–	–	12 (9%)	–	0
CCPR: pulmonary hypertension	–	–	–	–	0	–	v
CCPR: arrhythmia	–	–	28 (14.4%)	–	–	–	11 (17%)
CCPR: valve disease	–	–	–	2 (1%)	–	–	–
CCPR: myocarditis	2 (1.8%)	–	0	0	2 (2%)	–	–
CCPR: hypothermia	–	–	–	–	1 (1%)	–	–
CCPR: sepsis	–	–	–	–	1 (1%)	–	–
CCPR: brain hemorrhage	–	–	–	–	2 (2%)	–	–
CCPR: neurologic	–	–	–	–	–	–	1 (2%)
CCPR: electrolyte/toxin	–	–	–	–	–	–	0
CCPR: intoxication	–	–	–	–	–	–	0
CCPR: cardiomyopathy	–	–	7 (3.6%)	5 (4%)	6 (5%)	–	–
CCPR: post-cardiotomy	0	–	–	–	–	–	–
CCPR: bleeding	–	–	–	–	0	–	–
CCPR: type A aortic dissection	–	–	–	–	2 (2%)	–	–
CCPR: sarcoidosis/genetic mutation	–	–	–	–	–	–	1 (2%)
CCPR: unknown	13 (11.5%)	–	43 (22.2%)	67 (43%)	12 (9%)	100%	3 (4.7%)

ACS: acute coronary syndrome, CAD: coronary artery disease, CCPR: conventional cardiopulmonary resuscitation, ECPR: extracorporeal cardiopulmonary resuscitation.

No study reported on patient management stratified for (presumed) etiology of the arrest.

### Clinical outcomes

Survival and neurologically intact survival were assessed at different time points across studies. Reported survival to hospital discharge in the ECPR cohorts varied widely, ranging from 14.3% to 50% (Table 5). Neurologic intact survival at discharge was reported in another subset of studies and was observed in 20–90% of patients (Table 5). The included prospective studies did not report on neurologically intact survival rates at the time of discharge (Table 6). One study did not directly compare ECPR to CCPR but instead stratified outcomes by initial rhythm (shockable vs non-shockable), demonstrating a clear survival advantage for patients with shockable rhythms treated with ECPR [34].

**Table 5:** Complications and outcomes of included retrospective studies.

Author	Maekawa [18]	Lin [19]	Shin^2013^ [20]	Kim [21]	Siao [22]	Patel [23]	Cesana [24]	Han [25]	Mandigers [26]	Patricio [27]	Shin^2020^ [28]	Bartos [31]–	Choi [30]
Complication during/following ECPR	30 complications	–	–	16 (36.4%)	–	–	–	–	bleeding 74%, CVVH 37%, bacteriemia 28%	massive bleeding 57 (71%)	7 cases, 14 complications	–	–
Survival ECPR	17 (32.1%) discharge*, 15 (28.3%) 3 months*	29.1% (discharge)	29 (34.1%) in–hospital, 26 (30.6%) 6 months*	32 (58.2% 24 h)*, 8 (14.5% 3 months)	10 (50%) to discharge and 1 year	39.9% to discharge	13 (21%) discharge*, 12 (19%) 1 year	24% to discharge*	5 (26%) ICU survival*	18 (23%) ICU discharge#	10 (33.3%)	–	69/484 (14.3%) to discharge$
Neurologic intact survival ECPR	8 (15.4%)*	–	24 (28.2%) discharge, 24 (28.2%) 6 months*	8 (14.5% 3 months)*	8 (40%) 1 year*	–	92% discharge	20% to discharge*	4 (21%)*	17 (21%) 3 months	8 (26.7% 1 month)/10 (33.3% 6 months)*	52 (39%)	48/484 (9.9%)$

Survival CCPR	7(6.4%) discharge / 5 (4.6) 3 months	22.2% (discharge)	39 (12.1%) in-hospital, 35 (10.9%) 6 months	138 (31.% 24 h, 36 (8.1% 3 months)	11 (27.5%) to discharge, 10 (25%) 1 year	42.8% to discharge	49 (58%) discharge, 48 (56.4%) 1 year	9% to discharge	1 (5%) ICU survival	14 (18%) ICU discharge	6 (15.0%)	–	4707/117907 (4.0%)
Neurologic intact survival CCPR	3 (2.8%)	–	25 (7.8%) discharge, 24 (7.5%) 6 months	36 (8.1% 3 months)	3 (7.5%) 1 year	–	94% discharge	5.6% to discharge	0	9 (11%) 3 months	2 (5% at 1 and 6 months)	148 (23%)	1568/117907 (1.3%)

CCPR: conventional cardiopulmonary resuscitation, ECPR: extracorporeal cardiopulmonary resuscitation.

*Statistically significant difference between ECPR and CCPR groups.

^#^Statistically significant difference between ECPR and CCPR groups in log–rank test.

^$^Statistically significant in entire cohort, not significant in matched cohort.

**Table 6:** Complications and outcomes of included prospective studies.

Author	Chen [31]	Chou [32]	Sakamoto [33]	Nakashima [34]	Belohlavek [8]	Yannopoulos [7]	Suverein [17]
Complication during/following ECPR	–	No major complication related to ECPR	–	89 (36%)	79 (number of complications)	166 (number of adverse events, patients had at least one; also, aspiration and rib fractures were included)	33 (47%) patients with >1 event, mean per patient: 1.4 ± 0.9
Survival ECPR	17 (28.8%) to discharge*, 36 (78.3%) 1 year*	15 (34.9%) (unclear timeframe)#	177 (68.1% 24 h)	55 (22%) 6 months	–	6 (43%) discharge, 6 (43%) at 3 and 6 months*	–
Neurologic intact survival ECPR	14 (23.7%) at discharge*, 9 (15.3%) at 1y	–	32 (12.3%) 1 month*, 29 (11.2%) 6 months*	29 (11.7%) 6 months	38 (30.6%) 30 days*, 39 (31.5%) 6 months	CPC score at discharge 2.5 (0.5), at 6 months 1.16 (0.4)	14 (20% 30 days, 12 (18%) 3 months, 14 (20%) 6 months

Survival CCPR	14 (12.3%) to discharge, 40 (87%) at 1 year	5 (21.7%)(unclear timeframe)	37 (19.1% 24 h)	6 (3.8%) 6 months	–	1 (7%) at discharge, 0 at 3 and 6 months	–
Neurologic intact survival CCPR	12 (10.6%) at discharge, 10 (8.9%) at 1y	–	3 (1.5%) 1 month, 5 (2.6%) 6 months	4 (2.6%) 6 months	24 (18.2%) 30 days, 29 (22%) 6 months	CPC score at discharge 4, at 6 months NA	10 (16%) 30 days, 9 (14%) 3 months, 10 (16%) 6 months

CCPR: conventional cardiopulmonary resuscitation, ECPR: extracorporeal cardiopulmonary resuscitation, NA: not available.

^*^Statistically significant difference between ECPR and CCPR groups.

^#^Statistically significant difference between ECPR and CCPR groups in log-rank test.

Reporting on complications was limited. Bleeding was the most frequently described adverse event, with incidence rates as high as 74% in some cohorts (Table 5).

## Discussion

This mapping review highlights the current landscape of ECPR literature over the past two decades, revealing important trends and significant gaps in both clinical reporting and methodological rigor. Despite an exponential increase in the number of published studies, high-quality evidence demonstrating a consistent and meaningful survival benefit of ECPR – particularly with favorable neurological outcomes – remains limited. Survival following ECPR remains low, reportedly as high as 30%, reflecting both the critical nature of the patient population and persistent challenges in patient selection, timing, and support strategies [5].

To date, most published studies are observational, with relatively few prospective or randomized controlled trials. Only three randomized trials have enrolled patients specifically treated with ECPR, collectively including just 209 individuals. While post-hoc analyses from these trials have yielded some optimistic findings, the lack of high-quality, large-scale data continues to limit confidence in ECPR’s efficacy, especially regarding neurologically intact survival [35]. In addition, variability in practice and inconsistent initiation timing thresholds hinder broader implementation. Notably, no Cochrane meta-analysis has been published to date, and clinical adoption remains conservative in the absence of definitive evidence-based guidelines.

This focused evaluation identifies three key domains for advancement in ECPR practice: patient selection criteria, mechanistic insights into the pathophysiology, and the optimal timing of ECPR initiation. Together, these domains form a proposed framework to guide multidisciplinary collaboration with the goal to aid decision-making, reduce variability in care, and ultimately improve outcomes in critically ill patients and responsible use of extracorporeal resources in these patients.

### Patient selection

Timely and accurate identification of patients most likely to benefit from ECPR remains one of the greatest challenges in clinical practice. Current criteria are empirical and inconsistent. Prolonged decision-making allows ongoing hypoxia and ischemia to cause irreversible injury, with each minute of delay decreasing survival probability.

Although ELSO suggested general inclusion criteria – including age < 70 years, witnessed arrest, and shockable rhythm (ventricular fibrillation/pulseless VT/pulseless electrical activity) – these are largely consensus-based and not validated in prospective studies [36]. While more stringent selection (e.g., witnessed arrest, early CPR, end-tidal CO_2_ >10 mmHg, recurrent signs of life) is proposed to improve outcomes, this directly results in low overall eligibility rates, with merely 5–10% of OHCA cases [36]. This is in line with the meta-analysis by Karve, showing that more selection criteria and shorter low-flow times correlate with improved neurologic survival [37]. A recent systematic review focusing on eligibility criteria for ECPR also showed marked variability in patient selection strategies in the literature [38]. Moreover, previous studies have been limited by small sample sizes and heterogeneous populations, hindering subgroup analysis.

Etiology plays a critical role in outcomes, though many studies do not clearly report arrest cause, and some rely on presumed cardiac origins without confirmatory diagnostics. As shown in Tables 3 and 4, inconsistent etiological reporting impedes the development of stratified treatment protocols. It is likely that patients with specific causes of arrest (e.g., acute coronary syndrome or hypothermia) may derive more benefit from ECPR than others (e.g., pulmonary embolism), although clear evidence is still lacking [11, 34].

Future trials should prioritize phenotyping and prospective stratification to guide patient selection and personalize management strategy, incorporating clinical markers (e.g., end-tidal CO_2_, signs of life), comorbidities, and frailty assessments to define eligibility and maintain feasibility.

*Pathophysiology*

Understanding the underlying pathophysiology of refractory cardiac arrest is essential to optimizing ECPR application. Following circulatory collapse, generalized hypoxia initiates a cascade of metabolic derangements, including a shift to anaerobic metabolism, lactic acidosis, and adenosine triphosphate (ATP) depletion [39]. This leads to failure of the sodium-potassium-ATPase pump, resulting in ion imbalances, cytotoxic cerebral edema, and early neuronal injury. Excessive intracellular calcium further drives glutamate release, mitochondrial dysfunction, and reactive oxygen species (ROS) production, all contributing to irreversible cell death and multi-organ failure [39]. Hypoxia-induced endothelial dysfunction also primes the vasculature for ischemia-reperfusion injury. Upon reperfusion, additional damage – also referred to as the so-called “oxygen paradox” – can occur, involving ROS release, cytokine storm, and microvascular thrombosis, exacerbating cerebral edema, blood-brain barrier disruption, and coagulopathy [39–41]. To date, research on ischemia-reperfusion injury during ECPR is limited, and either descriptive in nature or originating from animal-derived data.

Extrapolating our current understanding of pathophysiologic mechanisms involved during low-flow states, application of high oxygen delivery and pump flow may appear intuitive, even though emerging data challenge this approach. Several studies suggest that supraphysiological oxygenation may even worsen neurological outcomes [42, 43]. Chang et al. reported a large range of PaO_2_, 77–220 mmHg (or 10–29 kPa), to be associated with more favorable neurologic outcomes following ECPR [44]. Nevertheless, most studies do not report oxygenation targets, MAP goals, or perfusion strategies, which not only limits comparability but also hinders evaluation of the association between ECPR strategy and outcomes in specific populations (Tables 1 and 2). Similarly, details on inotrope or vasopressor dosing, IABP use, and circuit characteristics are inconsistently provided.

An emerging area of interest within ECPR is the use of adjunctive therapies to promote myocardial recovery and facilitate conversion from malignant tachyarrhythmias to normal sinus rhythm. Among these, potassium-induced cardioplegia, traditionally employed during cardiopulmonary bypass to achieve electromechanical arrest, might be a promising adjunctive therapy in preclinical settings. Potassium-induced cardioplegia is hypothesized to act as a myocardial “reset”, enabling controlled reactivation of electrical activity and thereby promoting rhythm conversion, and may represent a valuable adjunct in the ECPR toolkit, particularly in cases of refractory ventricular tachyarrhythmias. A porcine model demonstrated that potassium-induced cardioplegia could achieve safe and effective conversion, while a rat model suggested that potassium-based cardioplegia helps to prevent diastolic dysfunction following prolonged arrest [45, 46]. Clinical data in humans are lacking, and important questions remain regarding optimal dosing, timing, safety, and clinical efficacy. At present, its use should be considered investigational or reserved for compassionate use in highly selected patients. These knowledge gaps underline the urgent need for randomized trials to guide the development of tailored ECPR strategies, which may need to vary based on arrest etiology, patient age, or comorbid conditions.

Since current knowledge is largely extrapolated from ECMO literature, the general understanding of perfusion-related organ injury during ECPR remains superficial. Translational studies are needed to delineate optimal post-initiation management of ECPR patients, including oxygen and CO_2_ targeting, anticoagulation protocols, and mitigating ischemia-reperfusion injury [13].

### Initiation timing

Among all determinants of outcome, initiation timing is arguably the most critical. During CCPR, only 25% of normal cardiac output and 4% of coronary perfusion are achieved under ideal conditions [47, 48]. Cerebral perfusion is even more limited, estimated at 2–11% of normal flow [49]. These low-flow states quickly lead to oxygen debt, acidosis, and cellular injury. Clinical studies consistently show that prolonged no-flow and low-flow durations are associated with worse outcomes, i.e., a decreased chance of return of spontaneous circulation and survival. Multiple observational studies and guidelines suggest ECPR initiation within 60 min of arrest (“low-flow interval”) to maximize favorable outcomes [50]. Consensus often recommends cannulation after 10–20 min of failed CCPR, before reaching 60 min of low-flow time [36]. Trials like INCEPTION, EROCA, ARREST, and others report significant variability in response times [7, 17, 51]. Prolonged initiation intervals well above 60 min were linked to lower survival, while networks achieving a shorter timeframe, closer to 50 min of low-flow time, showed better outcomes [17, 52]. In line with those reports are study data from Bartos and colleagues, which suggests that beyond 40 min, the accumulation of lactic acid and resulting acidemia substantially reduces the likelihood of neurologically intact survival [29]. While no universally accepted cutoffs exist, ELSO recommends <5 min of no-flow and < 60 min of low-flow time [36].

Achieving rapid ECPR initiation requires not only advanced prehospital logistics and continuous team readiness, but is also reliant on early recognition of cardiac arrest and immediate and sufficient bystander CPR. Public health efforts in qualitative CPR training for the general population and broad deployment of automated external defibrillators should be considered essential components of an integrated ECPR strategy [53].

## Integration

The aforementioned domains are interdependent, as pathophysiologic understanding should inform candidate selection and initiation timing. Future research must focus on integrated, randomized controlled study designs that combine biomarker-driven pathways, algorithm-based selection tools, and optimized initiation protocols. ECPR programs should prioritize rapid deployment, standardized management strategies, and rigorous data collection. In addition to the need for substantial resources and system-level coordination – including prehospital protocols, bystander CPR, and high-volume ECPR centers –, large-scale prospective trials and implementation studies are needed to refine patient selection and improve both survival and neurologic outcomes.

## Conclusion

Over the last two decades, ECPR has evolved, yet considerable heterogeneity persists in patient selection, management protocol, and involved timelines. Future research must integrate these three components in unified trials. This approach is crucial to optimize neurologic outcomes, reduce futile interventions, and justify ECPR’s widespread use. Nevertheless, ECPR should be considered a highly specialized intervention to be applied at experienced centers with multidisciplinary expertise.

## Data Availability

This study did not generate or analyze any new datasets. All materials and sources referenced in the manuscript are publicly accessible and are fully cited in the article's reference list.
